# Gut Bacterial Microbiota and its Resistome Rapidly Recover to Basal State Levels after Short-term Amoxicillin-Clavulanic Acid Treatment in Healthy Adults

**DOI:** 10.1038/s41598-018-29229-5

**Published:** 2018-07-25

**Authors:** Chad W. MacPherson, Olivier Mathieu, Julien Tremblay, Julie Champagne, André Nantel, Stéphanie-Anne Girard, Thomas A. Tompkins

**Affiliations:** 1 Lallemand Health Solutions Inc., 6100 Royalmount Avenue, Montreal, Quebec H4P 2R2 Canada; 2 National Research Council Canada, Energy, Mining and Environment, 6100 Royalmount Avenue, Montreal, Quebec H4P 2R2 Canada; 3 National Research Council Canada, Genomics, 6100 Royalmount Avenue, Montreal, Quebec H4P 2R2 Canada

## Abstract

Clinical effects of antimicrobials and probiotics in combination have been reported, however, little is known about their impact on gut microbiota and its resistome. In this study 16S rRNA gene amplicon, shotgun metagenomics sequencing and antibiotic resistance (ABR) microarray were used on fecal samples of 70 healthy participants, taken at four time points in probiotic (*Lactobacillus rhamnosus* R0011 and *Lactobacillus helveticus* R0052) and placebo groups to profile the gut bacterial microbiota and its resistome following administration of amoxicillin-clavulanic acid for one week. Significant shifts in microbiota family composition caused by the antimicrobial in both groups that included decreases in the proportion of *Lachnospiraceae*, *Coriobacteriaceae* and unidentified *Clostridiales*; and notable increases for the proportion of *Enterobacteriaceae*, *Bacteroidaceae* and *Porphyromonadaceae* compared to baseline levels. Resistome showed a corresponding enrichment of ABR genes compared to baseline from such classes as aminoglycosides and beta-lactams that were linked, by *in silico* inference, to the enrichment of the family *Enterobacteriaceae*. Despite perturbations caused by short-term antibiotic treatment, both gut microbiota and resistome showed prompt recovery to baseline levels one week after cessation of the antimicrobial. This rapid recovery may be explained by the hypothesis of community resilience.

## Introduction

It is widely acknowledged that the human gut microbiota is a complex microbial community implicated in a large number of beneficial host functions that include food processing (e.g., dietary fiber and polyphenols), enhancement of the intestinal epithelial barrier function, protection against pathogens, and development and modulation of innate and adaptive immune responses of various cell types of the gut-associated lymphoid tissue^1–3^. Thus, because of the vital role that the gut microbiota has on host well-being, any dysbiosis or perturbations in gut microbiota composition can consequently have detrimental impacts on human health that may ultimately lead to additional acute or chronic disease states^4,5^.

Recent studies have reported the impact of antibiotics on microbiota composition and the damaging consequences of the use of antibiotics on host well-being^4,6–8^. Raymond *et al*. addressed the observation from many microbiota studies of the high inter-individual diversity of the gut microbiota and evaluated how the initial microbiota composition was impacted by antibiotics^6^. In their study Raymond and co-workers administered cefprozil to 18 healthy participants and analyzed stool samples by shotgun metagenomics sequencing and concluded that the microbiota of healthy adults could be altered in a specific, reproducible and predictable manner^6^. This study also observed an increase of *Lachnoclostridium bolteae*, the emergence of *Enterobacter cloacae* and the detection of resistance genes that were previously not detectable before antibiotic treatment^6^.

The use of broad spectrum antibiotics and antimicrobials, such as ampicillin or amoxicillin-clavulanic acid, have been widely established to cause community-wide microbiota perturbations, leading to the repression of protective microbiota while promoting the growth of harmful indigenous bacteria that may result in the manifestation of clinical symptoms of Antibiotic-Associated Diarrhea (AAD) in susceptible individuals^4,9,10^. The combination of a β-lactam class of antimicrobial, amoxicillin, combined with a β-lactamase inhibitor, clavulanic acid, has been characterized to have bactericidal effects against bacteria that secrete β-lactamase^11,12^. This antimicrobial combination has also been reported, in a meta-analysis of 12 randomized placebo-controlled trials, to induce AAD in 10–25% of individuals^13^.

Despite the perturbations of microbiota by antibiotics, only a few studies have studied the short and long term impact of antibiotics both on the gut microbiota and its reservoir of antibiotic resistance genes known as the gut resistome^14,15^. One such study, looking at the effects of clarithromycin and metronidazole treatment on gut microbiota of three participants, reported dramatic shifts in microbiota diversity one week after antimicrobial treatment that eventually recovered to initial baseline states^16^. However, four years after antimicrobial use they were still able to detect high levels of the macrolide resistance gene *erm(B)*. According to the authors, this was indication that once selected, the gene can persist for extended periods^16^. Pérez-Cobas *et al*. evaluated the effect of antibiotics with different modes of actions for their impact on microbiota composition, as well as gut resistome, on four participants^4^. They reported that the antibiotic class and its mode of action had important contributing functions in modulating gut microbiota composition, while increasing the gut resistome^4^. Raymond and co-workers reported enrichment of ABR genes for 18 participants during antibiotic intake and found that resident microbiota returned to baseline levels 90 days after antibiotic treatment^6^.

Although these studies provide important insights on the impact of antibiotics on gut microbiota and resistome perturbations it must be noted that each study comes with notable limitations. It is well established that the intrinsic variation in the core microbiota composition across individuals (inter-individual variation) is important and it is therefore difficult to draw significant conclusions on studies investigating a limited number of participants^4,6^. Also, the absence of earlier sampling time points in these studies immediately following antibiotic use may have missed important details that would have provided the authors additional insights.

**Figure 1:**
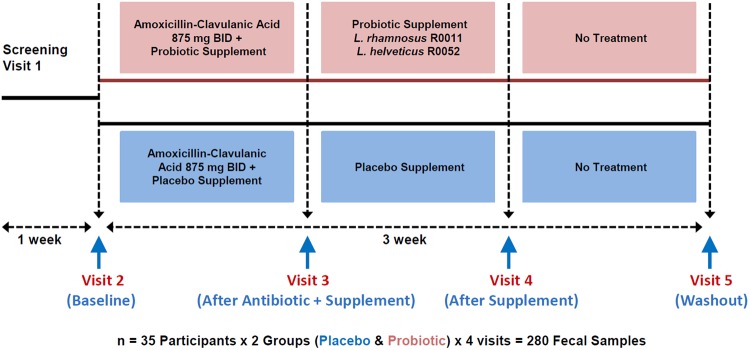
Overview of the clinical study design. There were 35 participants in probiotic and placebo groups totaling 70 participants. Fecal samples were taken at 4 time points for 16S rRNA gene amplicon sequencing and ABR microarray for a total of 280 DNA fecal samples that were analyzed.

A randomized, double-blind and placebo-controlled 10-week clinical trial (see Fig. 1 for study design) recently evaluated the impact of a probiotic combination (*Lactobacillus rhamnosus* R0011 and *Lactobacillus helveticus* R0052) on AAD following administration of amoxicillin-clavulanic acid treatment to 160 healthy adults^12^. The main finding of the study was that supplementation with the probiotic significantly (*p*-value < 0.05) reduced the duration of diarrhea-like defecations by one day^12^. In the present study, the objective was to further evaluate the impact of the antimicrobial treatment and probiotic supplementation on the gut bacteria microbiota and its resistome on a subset of 70 participants (35 per arm) from the original clinical study, in order to help explain the positive benefit outcomes found in the clinical study. For this purpose, we used 16S rRNA gene amplicon sequencing to profile the gut bacteria microbiota, a custom-designed antibiotic resistance (ABR) microarray to profile the gut resistome, and shotgun metagenomics sequencing to potentially associate antibiotic resistance genes with specific bacterial species.

## Results

### Gut microbiota composition and diversity

16S rRNA gene amplicon sequencing was used to profile the gut microbiota composition of fecal samples from our participants. To obtain a global image of the community dynamics in our microcosms, we computed alpha diversity (Observed Species/OTUs and Shannon indexes) and beta diversity (Weighted UniFrac) metrics (Supplementary Fig. S1–S2). As expected, alpha diversity was lower in samples of visit 3 after the amoxicillin-clavulanic acid administration. Beta diversity plots and Permanova tests (Supplementary Fig. S2) showed that samples cluster primarily by visit, indicating that this variable is the main driver in the formation of distinct communities. Interestingly, individuals tend to cluster together as well, suggesting the existence of a core microbiota profile unique to each individual, despite their treatments (Supplementary Fig. S3).

**Figure 2:**
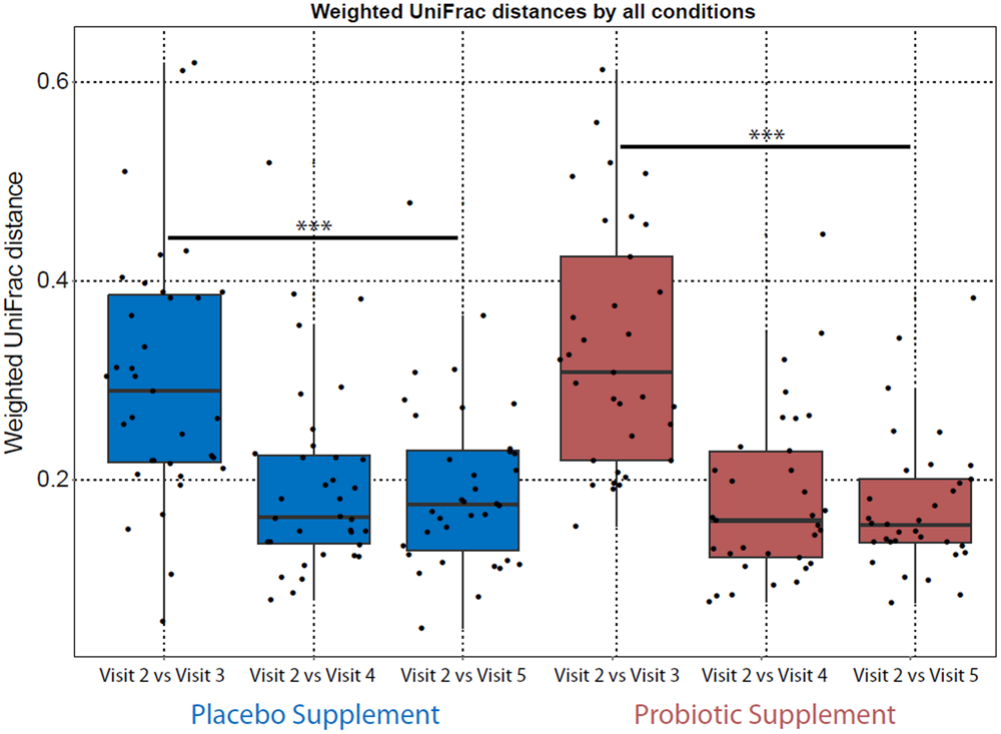
Weighted UniFrac distance (Beta diversity) analysis comparing how similar the microbiota is between the visits. Each dot represents one individual’s microbiota in visit 2 (baseline) that is compared to visits 3 (antimicrobial + probiotic/placebo supplement), visit 4 (probiotic/placebo supplement) and visit 5 (of wash-out). Two-way ANOVA followed by Tukey post-hoc test analysis showed that there was a significant difference for visit 3 compared to the other visits in both groups (***p-value < 0.001).

A two-way ANOVA analysis followed by a Tukey post-hoc test was done on weighted UniFrac distances to evaluate the microbiota profile similarity of each participant at each visit (visit 3, visit 4 and visit 5) compared to the baseline (visit 2) in both probiotic and placebo groups (Fig. 2). These results showed that the baseline (visit 2) and the antimicrobial treatment + probiotic/placebo supplement (visit 3) in both groups had more distant microbiota profiles (*p*-value < 0.001) when compared to probiotic/placebo supplement (visit 4) and washout (visit 5). There were no significant differences in UniFrac distances between visit 2 vs visit 4 and visit 2 vs visit 5, suggesting that bacterial microbiota found in these conditions were similar. More importantly, the results indicated that upon the amoxicillin-clavulanic acid intervention, there were shifts in the microbiota composition of both groups, but one week after the cessation of the antimicrobial, profiles returned to pre-treatment states in both groups and persisted thereafter in visit 5.

**Figure 3:**
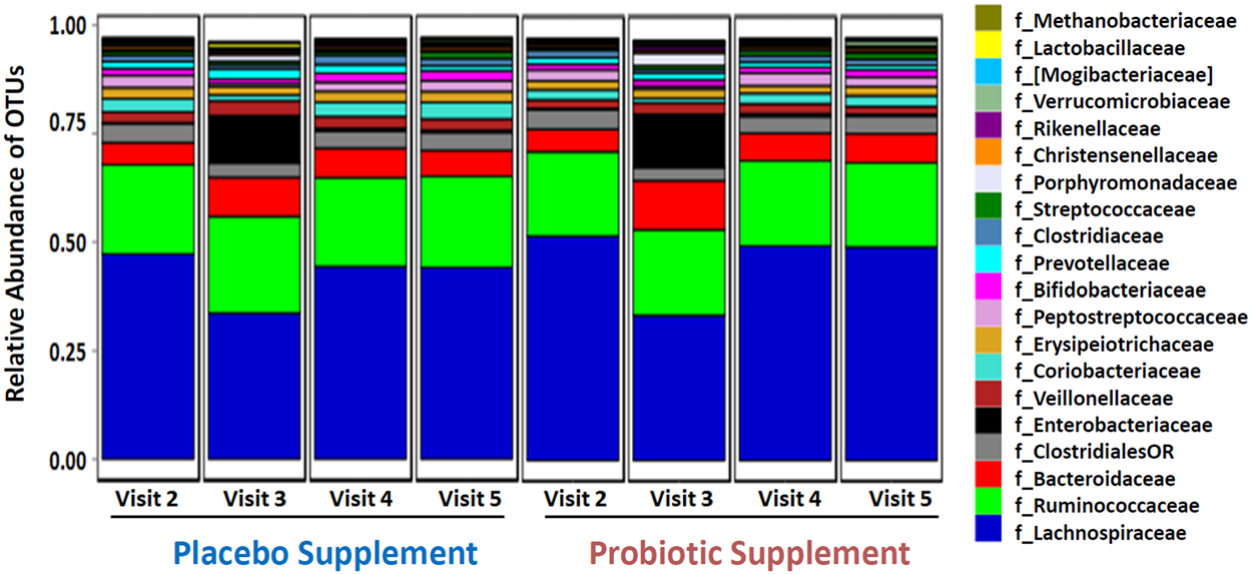
Community-wide microbiota composition profiling of the top 20 taxa down to the family level detected by 16S rRNA gene amplicon (V4 region) sequencing. The microbiota profiles in all 4 visits for both probiotic and placebo supplement represent the average relative microbiota composition abundance of all 35 participants in each visit of both arms of the study.

Further analysis of the 16 S rRNA amplicon sequencing data was performed to specifically evaluate major taxonomic differences in the bacterial microbiota composition between visits in both supplement groups. Figure 3 represents the average relative abundance of the top 20 taxa down to the family level for all the participants in each visit for both probiotic and placebo groups. The aim of this study was to evaluate what was the average microbiota composition of all the participants in each visit, with the full acknowledgement that there was significant inter-individual variation (Supplementary Fig. S3). Evaluating the average microbiota composition would allow for meaningful conclusions to be extracted, not on just one individual, but as a totality on both probiotic and placebo groups. Results in Fig. 3 showed that the most dominant taxa levels were for the family of *Lachnospiraceae*, followed by *Ruminococcaceae* and *Bacteroidaceae* in all the visits of both groups. Less dominant abundance levels also detected at the family level included *Veillonellaceae*, *Coriobacteriaceae*, *Erysipeiotrichaceae*, *Peptostreptococcaceae* and *Bifidobacteriaceae*.

More importantly, Fig. 3 showed that at visit 3, microbiota bacteria relative composition abundance levels were reduced for some taxa while enrichment was observed in other taxonomic families, but returned to similar baseline states in visit 4 and 5. This observation was consistent with the Weighted UniFrac distance analysis in Fig. 2 of the compositional shift of visit 3 compared to visit 2 and the subsequent return to pre-treatment levels. These results also suggest that administration of amoxicillin-clavulanic acid induced reproducible taxon relative abundance changes on microbiota composition, as well as exhibiting transient perturbations, as shown in both placebo and probiotic arms (Fig. 3).

The relative abundance of microbiota at visit 2 and visit 3 revealed many shifts in OTU relative abundance that were statistically (FDR < 0.01) significant (Fig. 3; Supplementary Table S5). For example, OTUs that were decreased in both groups in visit 3 that attained statistical significance included the families *Coriobacteriaceae*, *Peptostreptococcaceae*, *Lachnospiraceae*, and *Ruminococcaceae*, which all returned to levels similar to baseline in the last two time points after the cessation of the antimicrobial, as depicted in Fig. 3. Examples where major increases in relative OTU abundance levels from the baseline to antimicrobial + supplement were significant (FDR < 0.001) included the families *Bacteroidaceae* and *Enterobacteriaceae* (Fig. 3 and Supplementary Table S5), which also returned to baseline levels.

With the exception of minor OTU differences in the microbiota composition, the results revealed that there was a dynamic recovery from visit 3 to pre-treatment baseline composition levels in both the placebo and probiotic one week after amoxicillin-clavulanic acid treatment in visit 4 which continued to persist and stabilize into the wash-out period of visit 5. With regards to the impact of the probiotic on antimicrobial treatment, the results revealed no major compositional shifts on microbiota with probiotic, compared to placebo. However, there were some notable observations that may have been impacted by the probiotic supplement after antimicrobial treatment compared to placebo (visit 3).

In the probiotic supplement group at visit 3 there was a 4.4 fold-change increase (FDR < 0.0008) in the family of *Porphyromonadaceae* (specifically the genus *Parabacteroides;* Supplementary Table S5) compared to the baseline, whereas in the placebo supplement there was a lower fold-change increase of 3.0 (FDR < 0.01) when compared with the baseline (Fig. 3 and Supplementary Table S5). When directly comparing the results of both probiotic and placebo supplement groups of visit 3 there was a 1.76 fold-change increase (*p*-value < 0.014; FDR < 0.791) in the probiotic compared to the placebo supplement (visit 3).

### Relative qPCR of Enterobacteriaceae

**Figure 4:**
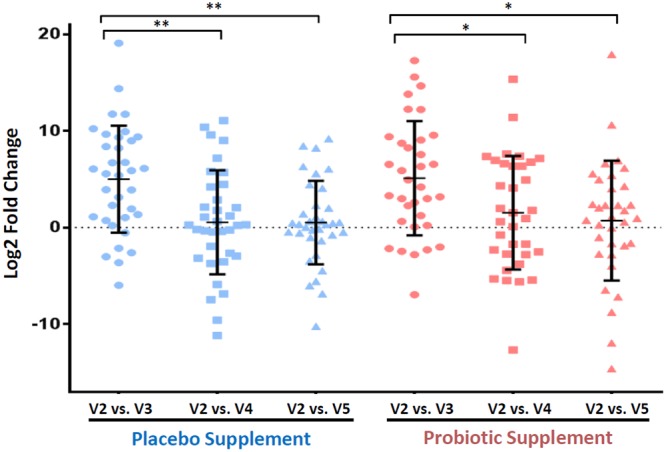
Relative quantitative PCR (qPCR) of the family of *Enterobacteriaceae* between the baseline visit 2 compared to antimicrobial + probiotic/placebo visit 3, probiotic/placebo supplement visit 4 and wash-out visit 5 in both groups. The reference gene used for relative qPCR was the 16S rRNA gene that targeted all bacteria. A Kruskal-Wallis test of One-way ANOVA (nonparametric) using Dunnett’s multiple comparisons analysis showed that there was an increase in the family of *Enterobacteriaceae* for visit 3 compared to visit 2 in both groups that was statistically significant compared to the other visits in both groups, thus confirming the results in the 16S rRNA gene amplicon sequencing for the enrichment of the family *Enterobacteriaceae*. *p-value < 0.05, **p-value < 0.01.

In order to confirm the 16S rRNA amplicon sequencing results, relative qPCR was performed on the same DNA from the 70 participants. The major compositional shift in the family *Enterobacteriaceae* in visit 3 due to antimicrobial treatment in both groups was chosen as the candidate family to confirm the results obtained in the 16S sequencing. The results in Fig. 4 indicated that the relative amount of the family of *Enterobacteriaceae* significantly (*p*-value < 0.05) increased in visit 3 relative to visit 2 compared to visit 4 and 5 for both probiotic and placebo groups, thus verifying the relative increase in the family of *Enterobacteriaceae* in visit 3 and its subsequent return to pre-treatment baseline levels.

### Shotgun sequencing of visit 3

**Figure 5:**
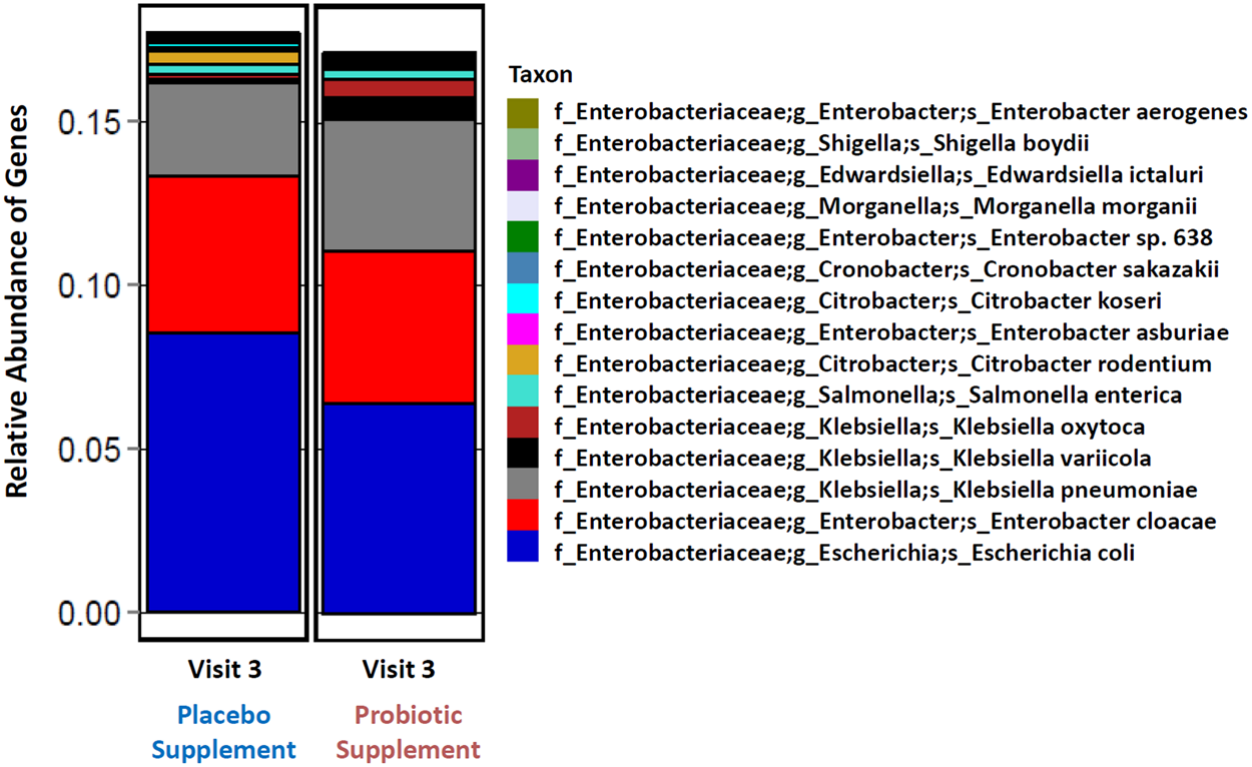
Relative abundance of top 15 taxa from shotgun metagenomics sequencing of pooled samples (n = 35) of visit 3 for both probiotic and placebo groups for the detection of species belonging to the family of *Enterobacteriaceae*. The purpose of the shotgun analysis was to determine if the enrichment of *Enterobacteriaceae* was due to commensal bacteria, opportunistic pathogens or the combination of both.

Shotgun metagenomic sequencing was performed in order to confirm our 16S rRNA gene amplicon profiles at a deeper taxonomic level to determine which specific genera and species were enriched within the *Enterobacteriaceae* group in visit 3 samples. All 70 participants for visit 3 were grouped together into two pools of samples for shotgun sequencing. The results in Fig. 5 showed that in both groups at visit 3, the enrichment of the *Enterobacteriaceae* family, which harbors a number of potentially opportunistic pathogens such as members of *Enterobacter*, *Klebsiella*, *Salmonella*, *Shigella* and *Escherichia* genera. However, it must be noted that most of the members of the *Enterobacteriaceae* family are normal inhabitants of the commensal flora. Further investigation would have to be performed to distinguish between the commensal bacteria and the opportunistic pathogens.

### Gut resistome analysis

**Figure 6:**
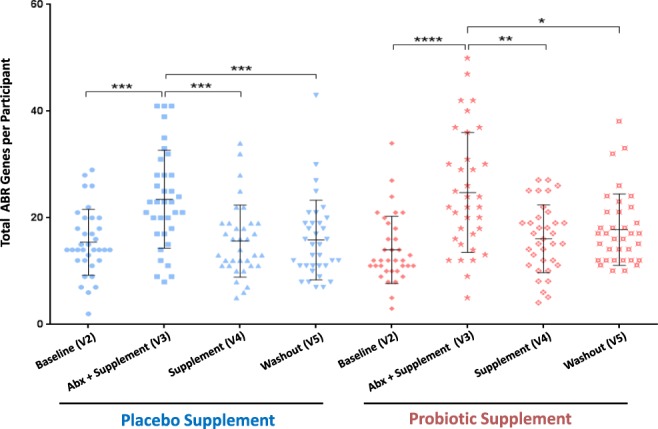
Total ABR genes whose DNA abundance increased by 2-fold or more after median normalization per participant in all visits of both probiotic and placebo groups. A Kruskal-Wallis test of One-way ANOVA (nonparametric) using Dunnett’s multiple comparisons test was used to evaluate the statistical differences of antimicrobial + treatment (visit 3) compared to the other visits in both arms of the study. *p-value < 0.05, **p-value < 0.01, ***p-value < 0.001, ****p-value < 0.0001.

ABR microarray analysis was used to evaluate the gut resistome by comparing the average number of detected ABR genes for all the participants at the different time points. In total there were 149, out of a possible 255, distinct ABR genes that were detected across all participants and visits. Moreover, the placebo group had 136 detected genes, whereas in the probiotic group 129 genes were detected. The results in Fig. 6 represent the total number of ABR genes having a 2-fold cut-off after median normalization for each participant. In all the visits the total number of ABR genes revealed inter-individual variability which is in agreement with the microbiota inter-individual variability for each participant. Moreover, Fig. 6 revealed that some participants had a greater increase in ABR genes during the amoxicillin-clavulanic acid intervention, whereas other participants did not respond as strongly. In comparing the baseline with the antimicrobial + supplement for both groups, there was a statistically significant (*p-*value < 0.001) average increase of ABR genes in visit 3 (Fig. 6). However, during the placebo/probiotic intervention (visit 4), one week after cessation of the amoxicillin-clavulanic acid, there was a statistically significant (*p-*value < 0.001) decrease of ABR genes, which stabilized after another week in the wash-out (visit 5).

### Enrichment of ABR gene class/type

**Figure 7:**
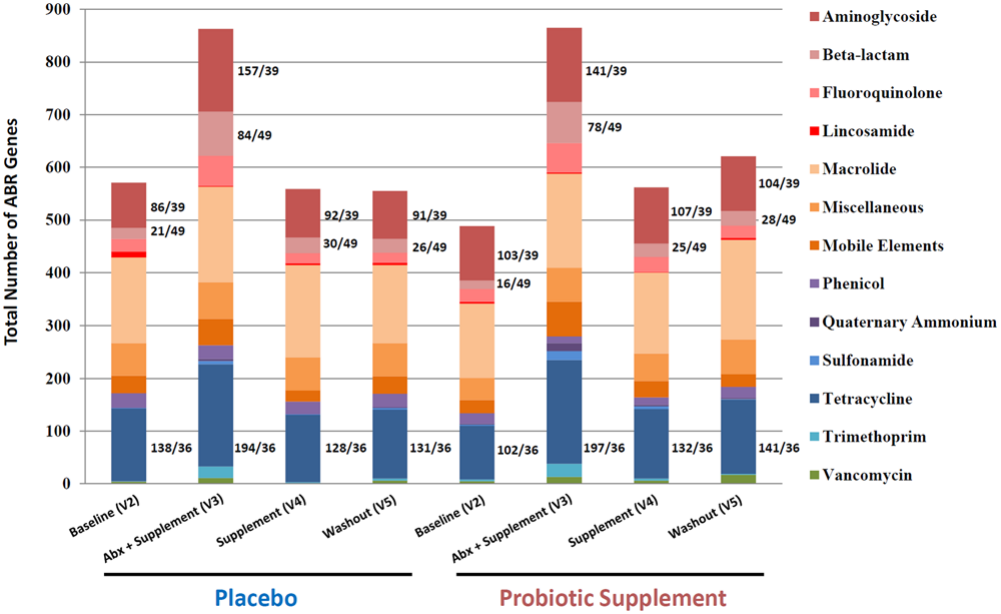
Gut resistome composition profile of the total number of ABR genes detected for all participants for each visit categorized into antibiotic classes or types. Major shifts or increases of specific ABR classes include aminoglycosides, beta-lactams and tetracycline. The ratio depicts the total number of ABR gene hits over the number of ABR gene probes on the custom-designed ABR microarray. For example, for the tetracycline class there were 36 distinct ABR probes on the microarray, but the results revealed there were many more tetracycline genes detected in all the visits of both probiotic and placebo arms. This is explained by the fact that there are many participants that carry the same tetracycline genes and, thus the frequency of occurrence of the same tetracycline genes are found throughout many participants in both arms of the study, as seen also with aminoglycosides and beta-lactams.

Further investigation into the gut resistome, both at an ABR class and type categorization level, revealed a number of notable insights. First, Fig. 7 showed that there was enrichment in the combined number of detected ABR genes in a few ABR classes. For example, in the baseline of the placebo group there was an increase in the total number of ABR genes for the class of aminoglycosides of 86 to 157 in the antibiotic + supplement which subsequently decreased to 92 in the supplement-only and stabilized at 91 genes after washout. Similar fluctuations were observed for genes encoding resistance to beta-lactams and tetracycline and the probiotic arm showed the same pattern for these 3 categories.

**Figure 8:**
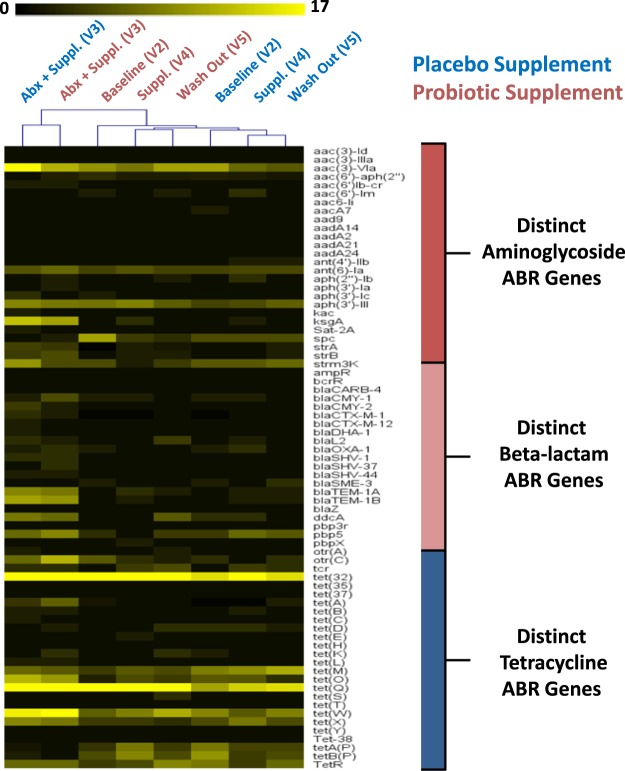
Hierarchical clustering heat map analysis for all the participants in each visit for both probiotic and placebo groups for the 3 major classes of aminoglycosides, beta-lactams and tetracycline. The heat map analysis shows the total number of distinct ABR genes for each class, as well as the high frequency of occurrence of some ABR genes that are common amongst the participants in each visit for both arms of the study.

The frequency of occurrence and enrichment in visit 3 of specific ABR gene classes for aminoglycosides, beta-lactams and tetracycline was further investigated to follow up the results obtained in Fig. 7 by performing two-dimensional hierarchical clustering analysis (Fig. 8). The heat map analysis illustrated the high frequency of occurrence of specific ABR genes in each of the visits, revealing the common ABR genes that were detected amongst all the participants. For example, Fig. 8 depicts how ubiquitous certain classes such as tetracycline and aminoglycosides were for a number of specific ABR genes. In addition, the results were not only consistent with previously reported results for the detection of *tet32*, *tetO*, *tetQ* and *tetW* in all the visits, but highlighted the high frequency of occurrence across all the participants. Moreover, the visits for placebo and probiotic groups clustered together in the following order of baseline, supplement and washout, showing similar ABR gene profiles amongst these visits. The results also emphasized a return to earlier pre-treatment baseline levels one week after antimicrobial cessation.

Furthermore, the clustering results from Fig. 8 also highlighted that the antimicrobial + supplement for both groups clustered together separately from the other visits, revealing a distinct profile of not only the enrichment of specific ABR genes, but also distinct ABR genes that were previously below the level of detection that are detected in visit 3 and that are either not detectable or at lower levels in visits 2, 4 and 5. Examples of distinct ABR genes (Fig. 8) that were uniquely enriched in visit 3 included: (1) *ksgA*, *strA* and *strB* for aminoglycosides, (2) *bla*_CARB-4_, *bla*_CMY-1_, *bla*_CMY-2_, *bla*_CTX-M-1_, *bla*_CTX-M-12_, *bla*_DHA-1_, *bla*_OXA-1_, *bla*_SHV-1_, *bla*_SHV-37_, *bla*_SHV-3_, *bla*_TEM-1A_ and *bla*_TEM-1B_, for beta-lactams, and (3) *tetA*, *tetB*, *tetC*, *tetK* and *tetL* for tetracycline. Conversely, a few ABR genes present in the baseline were absent in the antimicrobial + supplement, but were detected again in the supplement and washout. This included the aminoglycoside gene *AAC6-Ie*, the beta-lactam gene *sme-1* and tetracycline resistance genes *tetAP* and *tetBP* (Fig. 8).

### Enrichment ABR genes to *Enterobacteriaceae*

*In silico* analysis using the CARD database^17^ was performed on a selection of enriched ABR genes for the classes of aminoglycosides, beta-lactams and tetracycline (Fig. 8) in order to link specific ABR genes to the enrichment of the *Enterobacteriaceae* family in visit 3 (Fig. 3). For the class of aminoglycosides, three ABR genes, *ksgA*, *strA* and *strB*, were confirmed to be present in many genera of the *Enterobacteriaceae* family which included *Salmonella*, *Escherichia*, *Klebsiella*, *Shigella*, *Enterobacter* and *Citrobacter* (Supplementary Fig. S4). Similar to the aminoglycosides, there was a number of selected beta-lactam resistance genes that were enriched in visit 3 that included *bla*_CMY-1_, *bla*_CTX-M-1_, *bla*_CTX-M-12_, *bla*_DHA-1_, *bla*_SHV-1_, *bla*_SHV-37_, *bla*_TEM-1A_, *bla*_TEM-1B_, and *bla*_OXA-1_, that were all associated with a number of genera of the *Enterobacteriaceae* including *Salmonella*, *Escherichia*, *Klebsiella* and *Shigella*. It should also be noted that some of the aminoglycoside resistance genes, *strA* and *strB* and beta-lactam (*bla*_OXA-1_ only), were also found to be present in other Gram-negative bacteria that are not part of the family *Enterobacteriaceae*, such as the *Pseudomonas* genus. The tetracycline class had enrichment of the resistance genes *tetA*, *tetB*, *tetC*, *tetK* and *tetL* in visit 3, however, according to the CARD database, these genes were also present in many Gram-negative (e.g., *tetA*, *tetB*, *tetC*, *tetK* and *tetL*) and Gram-positive (e.g., *tetK* and *tetL* only) bacteria that were not exclusively present in the *Enterobacteriaceae* family.

**Figure 9:**
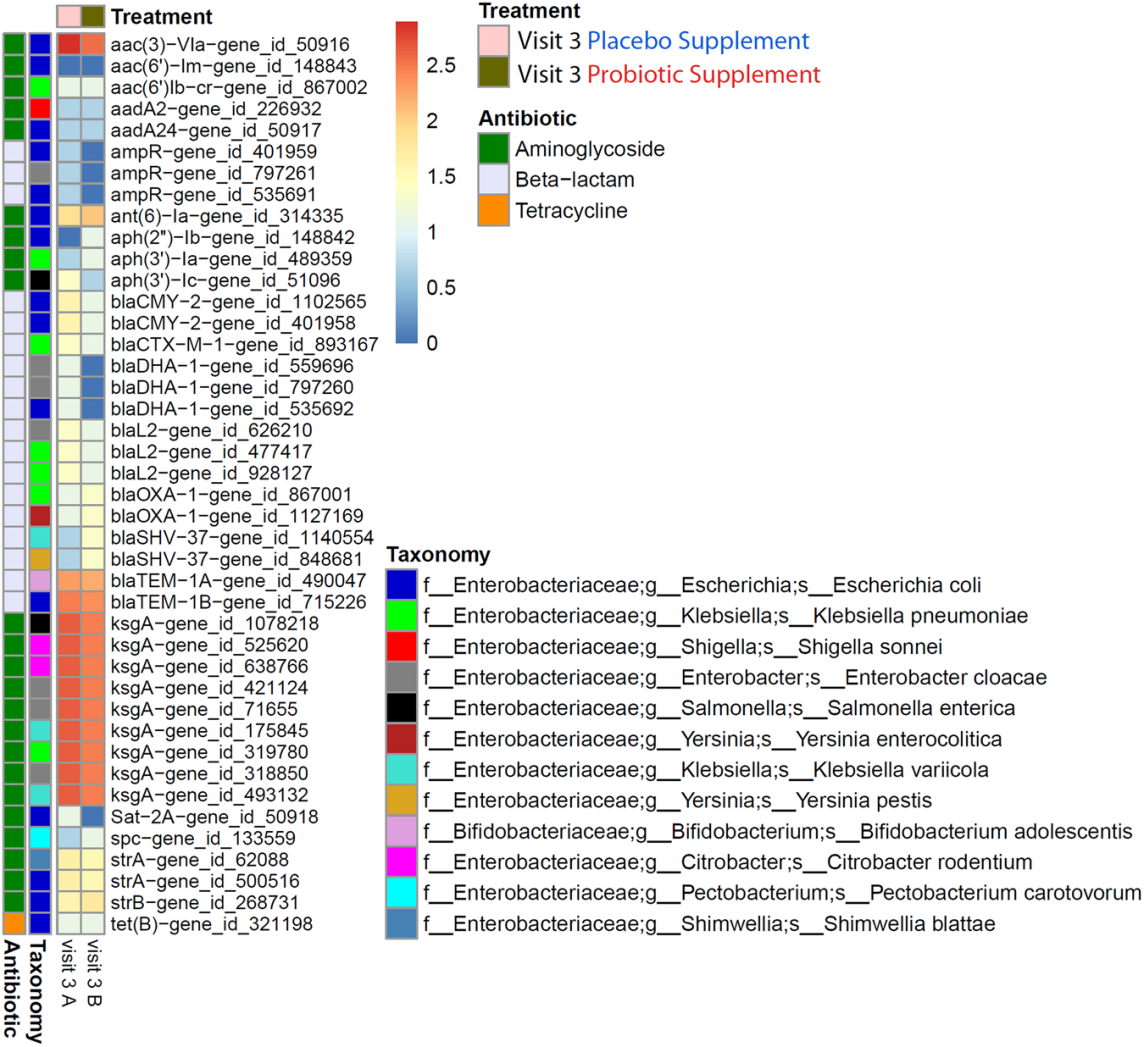
Shotgun metagenomics sequencing was used to confirm the enrichment of specific ABR genes in the classes of aminoglycosides, beta-lactams and tetracycline to the relative enrichment of specific species of the *Enterobacteriaceae* observed in the 16S amplicon sequencing results. ABR genes were linked to the enrichment of specific *Enterobacteriaceae* genes obtained with shotgun metagenomics sequencing, thus confirming the results found in the 16S sequencing, ABR microarray and *in silico* CARD analysis. The heat map scale represents a log2 (+1) transformed value of the abundance of ABR genes from shotgun metagenomics sequencing.

Finally, the shotgun metagenomics sequencing results obtained in Fig. 5 confirmed the detection of many species of the *Enterobacteriaceae* family which have been associated with an enrichment of specific ABR genes found in visit 3 (Fig. 8), thus providing further evidence to support the *in silico* predictions (Supplementary Fig. S4). The results in Fig. 9 revealed that the enrichment of specific ABR genes (Fig. 8) that was observed in visit 3 in both placebo and probiotic groups was associated with the enrichment of specific species of *Enterobacteriaceae*, thus linking the results that were obtained in the microbiota, microarray and *in silico* analyses.

## Discussion

The objective of this study was to examine the impact of the amoxicillin-clavulanic acid intervention and probiotic supplementation on the gut bacteria microbiota and its resistome on a subset of 70 healthy adult participants, in order to explain the positive outcome of probiotic supplement at reducing diarrhea-like defecation events^12^. Studies investigating the impact of antibiotics on the gut microbiota and resistome have reported inter-individual variations in microbiota before antibiotic treatment to prompt enrichment of ABR genes and shifts in microbiota composition and diversity during antibiotic treatment^6,9,18^. Other studies have reported that microbiota recovery after antibiotic treatment was in some cases transient and in other cases incomplete^16,19,20^. Work by Pérez-Cobas *et al*. evaluated the impact on the gut microbiota of four antimicrobials, including amoxicillin, with different modes of action on four human healthy participants before, during and after treatment with antimicrobials^4^. They reported that there were marked differences in the microbiota composition due to the specific antimicrobial, as well as notable differences in relative abundance of resistome genes by database search of metagenomes against the Antibiotic Resistance Genes Database^4,21^. It was also found that the composition between gut microbiota and resistome correlated to the surviving microbiota community^4^.

These studies provided important insights, however, one limitation of these investigations is the fact that there were very few participants in each of these studies (between 3–8 participants)^4,6,16,19,20^, thus making deductions on a population level incomplete and uncertain. A recent study by Raymond *et al*. using shotgun metagenomics sequencing of a larger cohort of 18 healthy human participants evaluated the impact of antibiotic treatment on both gut resistome and microbiota^6^. They concluded that antibiotic treatment can alter gut resistome and microbiome composition in a specific, reproducible and predictable manner and that enrichment of ABR genes and shifts in microbiome composition returned to baseline levels 90 days after antibiotic treatment cessation^6^. Interestingly, they also reported that a subgroup had enrichment in *E*. *cloacae* that was linked to lower microbiota diversity of the *Bacteroides* enterotype, suggesting that the initial composition of the microbiota ultimately determines the reshaping by antibiotics^6^.

Our results are also in accordance with the observation of inter-individual and transient variation in both gut microbiota composition (Figs 2 and 4) and resistome (Fig. 6), not only in the baseline of all the participants, but in all the visits of both probiotic and placebo groups. Our data shows that each participant harbors a unique core microbiota that reacts differently to the amoxicillin-clavulanic acid intake (Supplementary Fig. S3). In that regard, our study highlights the importance of having a large cohort of participants in order to infer statistically significant relevant conclusions, which to the best of our knowledge has not been previously reported on microbiota and its resistome. Furthermore, our results were also consistent with observations reported by Raymond *et al*. that in both the probiotic and placebo groups there were shifts in both microbiota composition and resistome incurred by antibiotic treatment that were specific, reproducible and predictable for a number of bacterial families and ABR classes/genes for both groups^6^. For example, in the microbiota results in Fig. 3 there were major relative shifts in such families as *Lachnospiraceae* (increase), *Bacteroidaceae* (decrease), unidentified *Clostridiales* and *Enterobacteriaceae* (increase). These trends were also observed in the resistome, especially with regards to specific ABR genes of the classes of aminoglycosides, beta-lactams and tetracycline (Figs 7 and 8), thus indicating and confirming that this antimicrobial treatment can induce specific, reproducible and predictable changes^6^. It must be noted that our ABR microarray does not comprise the full list of currently known and clinically relevant ABR genes to date and it is most likely that we do not have the complete picture of the gut resistome. Nevertheless, the ABR genes that we do have on our microarray clearly showed enrichment of ABR genes upon antimicrobial treatment and reversion back to baseline levels after antimicrobial cessation. It was also shown that there were no discernible shifts that were impacted by probiotic supplement on the administration of the antimicrobial combination with these genomic analyses, as the gut microbiota and resistome profiles were very similar in both groups. However, there are a few notable observations that may have been impacted by the probiotic supplement (visit 3) during antimicrobial treatment compared to placebo that warrant further investigation such as, 1) an increase in the family of *Porphyromonadaceae* (genus *Parabacteroides*) and 2) a relative decrease in the diversity of *Enterobacteriaceae* from the shotgun sequencing results (Fig. 5) in the probiotic group. Interestingly, Zackular and co-workers reported and hypothesized that *Porphyromonadaceae* may have an important protective role as anti-inflammatory mediators of gut health in a murine model of inflammation-driven colorectal cancer^22^. In another study, a randomized clinical study of 28 participants supplemented with the probiotic *Lactobacillus casei* Shirota to evaluate the impact on metabolic syndrome, reported the enrichment of the genus *Parabacteroides* of the *Porphyromonadaceae* family^23^, supporting the possibility that probiotic supplementation during antibiotic treatment may have increased the relative amount of the family *Porphyromonadaceae* in our study.

Although not a direct causality of probiotic supplement, the results obtained here, when taken together, provide new insights to investigate further the effect of this probiotic combination such as the impact of the family *Porphyromonadaceae* and inhibition of the growth of opportunistic pathogens. Furthermore, the major impact on gut microbiota and resistome composition was due to the combination of amoxicillin-clavulanic acid. However, according to Raymond *et al*. we must not exclude the possibility that the shifts that were observed may not only be due to the antibiotic treatment, but possibly other unknown fitness factors^6^.

More importantly, the major finding in our study was the prompt recovery or dynamic reversion to similar pre-treatment baseline levels of gut microbiota and resistome one week after amoxicillin-clavulanic acid cessation for both probiotic and placebo groups (with the exception of minor taxon differences). This rapid reversion has not been previously reported to our knowledge. Our results contradict other reports, where recovery of gut bacterial microbiota and resistome composition was either incomplete or was achieved in 28 to 90 days after antibiotic treatment^4,6,16,19,20^. It must be noted that other antibacterial agents, in addition to amoxicillin, were used in these studies such as cefprozil, clarithromycin, metronidazole, ciprofloxacin that are not directly comparable to amoxicillin-clavulanic acid in terms of the impact on gut microbiota. Thus, the difference may be explained by the type of antibacterial agent, the duration of the treatment or the health status of the study participants. Our study was done in healthy participants with a short-term antimicrobial intervention so a possible explanation for the prompt reversion to baseline levels may be explained by the hypothesis of microbiota community resilience^19,24^. This seems to be the case for short-term exposure to antimicrobials, but studies of long-term antibiotic use have reported that perturbations in gut microbiota and resistome can have either long-lasting irreversible effects or can persist for longer periods^16,18,25^. Moreover, according to Jakobsson *et al*. this highlights the proposal of restrictive antibiotic usage, in order to minimize the unknown consequences of long-term antibiotic use^16^.

It is interesting to note that Raymond *et al*. also reported inter-individual specific changes in resistance gene abundance, explaining that enrichment of important ABR genes may be below the level of detection and present at low abundance levels before beta-lactam treatment^6^. For example, they reported that beta-lactam resistance genes such as *bla*_OXA-1_ and *bla*_TEM-1_ were not detected before antibiotic treatment, but increased during antibiotic treatment and subsequently were not detected after antibiotic treatment^6^. These findings are also in agreement with our findings where specific ABR genes of beta-lactams such as *bla*_CMY-1_, *bla*_CTX-M-1_, *bla*_CTX-M-12_, *bla*_DHA-1_, *bla*_SHV-1_, *bla*_SHV-37_, *bla*_TEM-1B_, *bla*_TEM-1A_, and *bla*_OXA-1_ (Figs 8 and 9) were not detected in baseline, but were enriched after amoxicillin-clavulanic acid + supplement and subsequently decreased after supplement and washout in both groups. The explanation from Raymond *et al*. for the enrichment of certain ABR genes that are not detected in the baseline levels does not imply they are not present, but are, in fact, present at very low abundance levels that are simply below the level of detection^6^.

It has been reported in a number of studies that specific ABR genes are present in the gut microbiota of many individuals from different countries^15,16^. For example, the tetracycline genes of *tet32*, *tet40*, *tetO*, *tetQ* and *tetW* have been reported to be ubiquitously present in the gut microbiota of many individuals and to be the most abundant family of resistance genes^18,26^. These described results are also in agreement with our results for the genes *tet32*, *tetO*, *tetQ*, *tetW*, as well as *tetX*, which were found in all the visits of both arms. Moreover, the results in Fig. 7 for the class of tetracycline showed that there were 36 distinct ABR probes on our microarray, but there were many more tetracycline genes detected in all the visits of both placebo and probiotic arms. This is explained by the fact that there are many participants that carry the same tetracycline genes and, thus the frequency of occurrence of these same tetracycline genes is found throughout many participants in all the visits of both arms of the study.

We also observed specific and potentially important ABR genes such as the class of aminoglycosides and beta-lactams that were enriched after the antimicrobial + supplement, which were subsequently decreased, not detected or detected at very low levels after the supplement and washout. The enrichment of selected ABR genes in the aminoglycosides and beta-lactams was analyzed by the CARD database to deduce a possible link to the relative enrichment of microbiota families, thus connecting the two datasets. Interestingly, our *in silico* database search of enriched ABR genes had hits to the Gram-negative bacteria family of *Enterobacteriaceae* and in some cases specific genera of *Salmonella*, *Escherichia*, *Klebsiella*, *Shigella*, *Enterobacter* and *Citrobacter*. This not only validates the gut resistome and microbiota results for the enrichment of the family of *Enterobacteriaceae*, but implies that many of the members of this family harbor multiple ABR genes, potentially on plasmids, and thereby impart multidrug resistance.

Investigators have also reported on the enrichment of the *Enterobacteriaceae* family and have concluded that such enrichment may be due to a rise in pathogenic and opportunistic pathogens such as *Escherichia*, *Salmonella* and *Enterobacter*^4,6,27^. Raymond *et al*. also showed by shotgun sequencing increases in a subgroup of participants that were enriched with *E*. *cloacae* after exposure to antibiotic, as well as enrichment of beta-lactam resistance genes^6^. Although enrichment of the *Enterobacteriaceae* family may be due, in part, to the increase of pathogenic and opportunistic pathogens, such assumption may not be entirely accurate, or justified, as there are many commensal Gram-negative bacteria that belong to the *Enterobacteriaceae* family (e.g., strains of *E*. *coli*, and *E*. *cloacae*) that are normal constituents of the gut microbiota. To probe deeper into the *Enterobacteriaceae* family, shotgun metagenomics sequencing was also used to confirm the link of the enrichment of specific ABR genes to the possible enrichment of opportunistic pathogens. To this end, the shotgun results agreed with the *in silico* analysis (CARD and KEGG databases) that the enrichment of specific ABR genes may be linked to species within the *Enterobacteriaceae* family, namely *E*. *coli*, *E*. *cloacae*, and *K*. *pneumoniae*; three of the most abundant species found in the shotgun metagenomics data (Figs 5, 9 and Supplementary Fig. 4).

Overall, the results obtained in this study clearly showed that treatment with amoxicillin-clavulanic acid induced many community-wide shifts in gut bacterial microbiota and resistome composition. In both probiotic and placebo groups, enrichment of specific ABR genes of aminoglycosides and beta-lactams appeared to be associated with the enrichment of members of the *Enterobacteriaceae* family. Typically members of this family are normal intestinal residents, but some members may potentially be opportunistic pathogens. Moreover, perturbations in gut microbiota and resistome composition occurred in a specific, reproducible and predictable manner for both arms and the subsequent return to pre-treatment baseline-like levels one week after short-term antimicrobial treatment can be explained by the hypothesis of community resilience^19,24^. Lastly, further investigation into the impact of the probiotics on *Porphyromonadaceae* relative abundance may help explain the positive outcome of reducing the duration of diarrhea-like defecation events.

## Methods

### Study participants

Participants were described previously in Evans *et al*.^12^. The 160 participants were randomized into two arms. A randomization schedule was prepared using block randomization by an unblinded person at the study site who was not involved in the study assessment. All participants were healthy, free-living volunteers with a mean age of 34 years and a male to female ratio of 29:51. There were no statistical differences in the demographics and characteristics of the participants between the two arms. All participants received 1 capsule per day of the antimicrobial (875 mg of amoxicillin trihydrate and 125 mg of potassium clavulanate) for seven consecutive days simultaneously with the probiotic or placebo. A seven-day intervention was chosen as it is consistent with clinical use of this antimicrobial. It is typically used for 3–7 days for urinary tract infections (UTIs) or 7–10 days for respiratory infections.

### Human fecal samples

280 fecal samples were obtained from 70 healthy adults (35 participants per arm). The samples were from a subset of the two arms of the study reported by Evans *et al*.^12^. Both groups (probiotic combination of *Lactobacillus rhamnosus* R0011 and *Lactobacillus helveticus* R0052, and placebo) received one week intervention with amoxicillin-clavulanic acid. Four fecal samples were collected per participant at one week intervals that included: baseline (visit 2), antimicrobial + probiotic or placebo (visit 3), probiotic or placebo-only (visit 4) and wash-out (visit 5) as shown in Fig. 1. The randomized samples were sent in a blind fashion for DNA extraction and microbiota analyses.

### DNA extraction

Total DNA was isolated from approximately 250–350 mg of homogenized fecal samples using the QIAamp® Fast DNA Stool Mini Kit (Qiagen, Hilden, Germany) as per manufacturer’s instructions, with the following modifications. Two 0.05 M phosphate buffer washes prior to the addition of InhibitEX (Qiagen) and a 1 mm zirconia/silica bead beating step (~250–350 mg/tube, 4 m/s for 1 min × 3) before centrifugation of samples to pellet stool particles. DNA purity was assessed by 260/280 ratios. All DNA samples had ratios between 1.8–2.0, which were further processed for ABR microarray hybridization, relative-qPCR, 16S gene amplicon and shotgun metagenomics sequencing.

### Amplification and sequencing of the bacterial 16S rRNA gene

Libraries for sequencing were prepared according to Illumina’s “16 S Metagenomic Sequencing Library Preparation” guide (Part # 15044223 Rev. B), with the exception of using Qiagen HotStar MasterMix for the first PCR (“amplicon PCR”) and halving reagent volumes for the second PCR (“index PCR”). The template specific primers were (without the overhang adapter sequence): 515 f (5′-GTGCCAGCMGCCGCGGTAA-3′) and 806 R (5′-GGACTACHVGGGTWTCTAAT-3′). The first PCR (“amplicon PCR”) was carried out for 25 cycles with annealing temperatures of 55 °C. Diluted pooled samples were loaded on an Illumina MiSeq and sequenced using a 500-cycle MiSeq Reagent Kit v3.

### Shotgun sequencing of visit 3 pooled samples

Pooled samples were prepared from the 35 DNA extracts (1 per participant) for each arm and for visit 3. Total nucleic acids were recovered using a modified version of the hexadecyl trimethyl ammonium bromide (CTAB) method (Ausubel, 2002). The modifications were as follows: the incubation time for the TE/Lysozyme treatment was reduced substantially from one hour to 15 minutes and the temperature for this step was increased from 37 °C to 56 °C. The CTAB/NaCl incubation was followed by phenol/chloroform/isoamyl alcohol (25:24:1), then a chloroform/isoamyl alcohol extraction step. DNA was quantified using Quant-iT PicoGreen assay (Invitrogen, Life Technologies) and 1 ng of gDNA was used as a template to construct the sequencing library, using the Illumina Nextera XT library preparation protocol following the manufacturer’s instructions. However, the “Library Normalization” step was omitted and normalization was instead performed by pooling equal amounts of libraries after Quant-iT PicoGreen quantification. The quality of the pooled library was assessed using an Agilent 2100 Bioanalyzer with a High Sensitivity DNA Kit. (http://support.illumina.com/sequencing/sequencing_kits/nextera_xt_dna_kit/documentation.html). Libraries were sequenced on a HiSeq2500 system (Illumina) on a 2 × 125 bp configuration. Both pools contained 35 samples and yielded 227 and 238 million reads for pools A and B respectively, which makes an average of 6.66 million reads per individual (although no multiplexing was performed).

### Microbiota data analysis

Sequences were analyzed through the National Research Council’s (NRC, Montreal, Canada) rRNA short amplicon analysis pipeline as previously described^28,29^. Reads were QCed, paired-end assembled and clustered at 97% similarity. Resulting operational taxonomic units (OTUs) were assigned a taxonomic lineage using the Ribosomal Database Project (RDP) classifier (v2.5) with a custom Greengenes (v13_5) training set^28–31^. Taxonomic summaries and alpha (observed) and beta (weighted or unweighted UniFrac distances) diversity metrics and taxonomic classifications were computed using QIIME software^32^ and downstream analyses were done with in-house Perl and R scripts. A read count summary of the various steps of the analysis pipeline and metadata used to conduct the analyses are available in Supplementary Table S1–S2.

Shotgun metagenome data was analyzed using the NRC’s shotgun metagenomics pipeline as described^33^. Briefly, reads were quality controlled and assembled, after which genes were called from the contigs and annotated following the Joint Genome Institute’s (JGI) guidelines^34^. Quality filtered reads were mapped on assembled contigs to obtain abundance profiles, which were normalized to CPM (Counts Per Million) using EdgeR v3.10.2^35^. Metagenome assembly statistics and read counts summaries are available in additional Supplementary Table S3–S4. Identification of antibiotic resistance genes was done by doing a sequence homology search (BLASTn) using the online tools of the specific database. The microarray probes sequences were compared against the assembled metagenome contigs (blastn v2.2.29+). Alignment results were filtered to keep only probes having an e-value ≤ 1e-03.

### Relative qPCR for *Enterobacteriaceae*

Quantification of *Enterobacteriaceae* family levels in clinical fecal samples for validation of 16S rRNA gene amplicon sequencing were performed by relative quantitative PCR (qPCR) according to the MIQE guidelines^36^. Genomic DNA samples were amplified with the following *Enterobacteriaceae* family specific primers, ECO1456F CATTGACGTTACCCGCAGAAGAAGC and ECO1652R CTCTACGAGACTCAAGCTTGC^37^. The *Enterobacteriaceae* relative amounts were normalized with the 16S rRNA housekeeping gene amplified with the UniF (ACTCCTACGGGAGGCAGCAGT) and UniR (ATTACCGCGGCTGCTGGC) specific primer pair^38^. Individual 25 µL qPCR reactions containing 2.5 µL of a 1:5 gDNA dilution were performed with SYBR Select Master Mix (Thermo Fisher, cat. #4472908) and 300 nM primers (IDT, IA, USA) on an ABI 7300 Real-Time Cycler (Thermo Fisher, MA, USA). Cycling conditions were as follows: 50 °C and 95 °C both for 2 minutes each prior to 40 cycles of 95 °C 15 seconds denaturation, 60 °C 30 seconds annealing and 72 °C also 30 seconds for primer extension, generating a 193 or a 195 bp amplicon for *Enterobacteriaceae* or 16S rRNA gene, respectively. Amplification specificity was checked with a melting curve analysis and an agarose gel electrophoresis for both primer pairs. Additionally, primers were tested for amplification efficiencies by amplifying serial 10-fold dilutions of gDNA from an *E*. *coli* strain for *Enterobacteriaceae* family and a *Lactobacillus helveticus* R0052 for 16 S rRNA gene. The calculated efficiencies were 95.2% and 97.6%, respectively. The ΔΔCt method was used to determine the relative fold-change for each of the participants in the placebo and probiotic groups. A Kruskal-Wallis test of one-way ANOVA (nonparametric) using Dunnett’s multiple comparisons test was performed with GraphPad version 6 to determine the statistical significance.

### Antibiotic resistance microarray design

We used a custom-designed oligonucleotide-based DNA microarray containing a total of 370 probes targeting 254 unique antibiotic resistance (ABR) genes and 20 mobile elements (Lallemand Health Solutions Inc., Montreal, Canada). ABR microarray probes were selected from publications by Peterson *et al*. and Frye *et al*.^39,40^ and spotted on CSE-5321 sciEPOXY slides (Scienion, NJ, USA). A detailed list of the ABR genes and class categories on the microarray can be found in Table 1. The ABR genes and class categories were also confirmed by ResFinder^41^, Antibiotic Resistance Gene-ANNOTation (ARG-ANNOT)^42^ and Comprehensive Antibiotic Resistance Database (CARD)^17^.

**Table 1:** List of antibiotic resistance genes (ABR) and mobile elements on the custom designed ABR microarray according to class/type.

Antibiotic Classes/Types	Total Genes
**Aminoglycoside**	
*aac(3)-Ia*, *aac(3)-Id*, *aac(3)-IIa*, *aac(3)-IIIa*, *aac(3)-Iva*, *aac(3)-Via*, *aac(6′)-aph(2*″*)*, *aac(6′)-Ib-cr*, *aac(6′)-Im*, *aac6-Ii*, *aacA4*, *aacA7*, *aad9*, *aadA*, *aadA14*, *aadA2*, *aadA21*, *aadA24*, *aadA7*, *aadB*, *aadD*, *aadK*, *ant(4′)-IIb*, *ant(6)-Ia*, *aph(2*″*)-Ib*, *aph(2*″*)-Id*, *aph(3′)-Ia*, *aph(3′)-Ic*, *aph(3′)-IIa*, *aph(3′)-III*, *aph(3′)-IV*, *kac*, *ksgA*, *sat2A*, *sat4A*, *spc*, *strA*, *strB*, *streptomycin-3-kinase*.	n = 39
**Beta-Lactam**	
*ampR*, *bla1*, *bla2*, *blaB-13*, *blaB-2*, *blaCARB-1*, *blaCARB-2*, *blaCARB-4*, *blaCMY-1*, *blaCMY-2*, *blaCTX-M-1*, *blaCTX-M-12*, *blaDHA-1*, *blaFOX-2*, *blaIMP-2*, *blaIMP-4*, *blaKPC-3*, *blaL2*, *blaOXA-1*, *blaOXA-2*, *blaOXA-7*, *blaOXA-9*, *blaOXA-26*, *blaOXA-27*, *blaOXA-61*, *blaOXY-2–2*, *blaPER-2*, *blaROB-1*, *blaSHV-1*, *blaSHV-37*, *blaSHV-44*, *blaSME-1*, *blaSME-2*, *blaSME-3*, *blaTEM-1A*, *blaTEM-1B*, *blaTEM-116*, *blaVIM-2*, *blaZ*, *ccrA*, *ccrB*, *ddcA*, *mecA*, *mecI*, *pbp-1A*, *pbp3r*, *pbp5*, *pbpX*, *penA*.	n = 49
**Fluoroquinolone**	
*acrR*, *cme*, *cmeA*, *cmeB*, *cmeC*, *cmeR*, *cmr*, *emrA*, *emrB*, *mar*, *oqxA*, *oqxB*, *qnrA1*, *qnrB1*, *qnrB4*, *qnrD*, *qnrS1*, *qnrS2*,	n = 18
**Lincosamide**	
*carA*, *lmrA*, *lmrB*, *lnuA*, *lnuB*.	n = 5
**Macrolide**	
*ereA*, *ereB*, *ermA*, *ermAM*, *ermB*, *ermC*, *ermD*, *ermF*, *ermG*, *ermQ*, *ermT*, *ermX*, *ermY*, *lsaB*, *mefA*, *mphA*, *mphB*, *mphC*, *mreA*, *msrA*, *msrC*, *smpA*, *srmB*, *stpA*, *tlrC*, *ybit*.	n = 26
**Miscellaneous**	
*albA*, *ank*, *arr3*, *bcrR*, *ble*, *colE1*, *emtA*, *erfA*, *erfB*, *mdfA*, *mmr*, *mupR*, *nimA*, *nimB*, *nonR*, *norA*, *oleB*, *oleC*, *res*, *vph*.	n = 20
**Mobile Element**	
*int1(2)*, *int1(3)*, *int1(3′-CS)*, *int2*, *int2_TnsA*, *int3*, *int3_repC*, *IS1182*, *IS150*, *pAD1*, *pAM alpha 1*, *pBR322*, *pMMB66EH*, *pSLT*, *RP4 IncP*, *Tn925*, *tnpA*, *tnpM*, *trans*, *trans1*.	n = 20
**Phenicol**	
*cat*, *cat(pC194)*, *cat(pC221)*, *cat86*, *catA1*, *catA2*, *catA3*, *catB*, *catB2*, *catP*, *catQ*, *catS*, *cfr*, *cmlA1*, *cmlV*, *fexA*, *floR*.	n = 17
**Quaternary Ammonium**	
*qacEdelta1*, *qacJ*, *smr*.	n = 3
**Streptogramin B**	
*msrD*, *vatA*, *vatB*, *vatC*, *vatD*, *vatE*, *vatF*, *vgaA*, *vgbA*, *vgbB*.	n = 10
**Sulfonamide**	
*sul1*, *sul2*, *sul3*, *sulI*.	n = 4
**Tetracycline**	
*otrA*, *otrC*, *tcr*, *tet31*, *tet32*, *tet33*, *tet34*, *tet35*, *tet36*, *tet37*, *tet38*, *tet39*, *tetA*, *tetAP*, *tetB*, *tetBP*, *tetC*, *tetD*, *tetE*, *tetG*, *tetH*, *tetJ*, *tetK*, *tetL*, *tetM*, *tetO*, *tetQ*, *tetR*, *tetS*, *tetT*, *tetU*, *tetV*, *tetW*, *tetX*, *tetY*, *tetZ*.	n = 36
**Trimethoprim**	
*dfrA1*, *dfrA5*, *dfrA7*, *dfrA8*, *dfrA9*, *dfrA12*, *dfrA13*, *dfrA15*, *dfrA20*, *dfrC*, *dfrD*.	n = 11
**Vancomycin**	
*ddlA*, *vanA-A*, *vanA-Ao2*, *vanA-B*, *vanA-C*, *vanA-D*, *vanA-G*, *vanE*, *vanH-A*, *vanH-D*, *vanR-A*, *vanS-A*, *vanX-A*, *vanY-A*, *vanY-D*, *vanZ-A*	n = 16

### Microarray hybridization, scanning and quantification

200 ng of DNA extracted from fecal samples was Cy5-labelled with Bioprime® DNA Labeling System (Life Technologies) and purified using the QIAquick PCR purification kit as per the manufacturer’s instructions. Each Cy5-labelled DNA was added to hybridization buffer (20 µl DIG Easy Hybridization Buffer, 1 µl 10 mg/ml Yeast tRNA and 1 µl 10 mg/ml Salmon Sperm DNA) and hybridized for 18 hours at 50 °C followed by washing 3 × 1 × SSC/0.1% SDS at 42 °C and final wash in 0.1 × SSC at RT. All washes were performed for 6.5 min. After the final wash, slides were dried by centrifugation for 2 min at 2000 rpm. Slides were scanned using ScanArray 5000 instrument from Perkin-Elmer (Waltham, MA) and spot intensities were quantified using Quant Array.

### Microarray data analysis

Quantile normalization for each microarray and median normalizations for each gene were performed. A Kruskal-Wallis test of one-way ANOVA (nonparametric) with Dunn’s multiple comparisons test was used to determine the statistical difference between the total number of ABR genes for each participant in each of the visits for placebo and probiotic groups. Two-dimensional hierarchical heat map clustering analysis was performed with Multi-Experiment Viewer (MeV, version 4.9) for selected ABR gene classes for aminoglycosides, β-lactam and tetracycline for each of the visits (2–5) in both placebo and probiotic groups^43^. Selected ABR genes from the heat map analysis that were enriched in visit 3 for both placebo and probiotic groups for aminoglycosides and beta-lactams were linked to various members of the family of *Enterobacteriaceae* using the Comprehensive Antibiotic Resistance Database, CARD^17^ as shown in Supplementary Fig. S4.

### Data Availability Statement

All raw sequence reads for 16S rRNA amplicons sequencing have been submitted to the sequence read archive (NCBI) under accession SRP120170 under Bio Project PRJNA414540. Raw shotgun metagenomics pooled samples for visit 3 were submitted under BioSample accessions SAMN07795010 and SAMN07795011 under submission ID: SUB3134289. Additionally, we did not write new bioinformatics tools – only used existing ones as previously referred to in the methods section under microbiota data analysis.

### Ethics Approval and Consent to Participate

The original study/trial was previously performed in accordance with the ethical principles of the Declaration of Helsinki and its subsequent amendments (Clinicaltrials.gov identifier NCT01941160) as described in Evans *et al*.^12^. Additionally, this study was reviewed by the Therapeutic Products Directorate (TPD) and the Natural and Non-prescription Health Products Directorate of Health Canada, and approvals were obtained on 1 August 2013 from the TPD, Ottawa, Ontario. Research ethics board approval was obtained on 13 August 2013 from Institutional Review Board (IRB) Services, Aurora, Ontario. Informed consent was given by all participants in this study.

## Electronic supplementary material

Supplementary Figures S1-S4

Supplementary Table S5 Statistical Comparison OTUs

Supplementary Dataset Table S1-S4 16S Metadata
