# Supplementary material for: Gut Bacterial Microbiota and its Resistome Rapidly Recover to Basal State Levels after Short-term Amoxicillin-Clavulanic Acid Treatment in Healthy Adults
Source: Sci Rep. 2018 Jul 25;8:11192. doi: 10.1038/s41598-018-29229-5 (PMC6060159; doi:10.1038/s41598-018-29229-5)
Supplement: Supplementary file 1 — Supplementary Figures S1-S4 [file 41598_2018_29229_MOESM1_ESM.pdf]

Gut Bacterial Microbiota and its Resistome Rapidly Recover to Basal State Levels after Short-term Amoxicillin-Clavulanic Acid Treatment in Healthy Adults

Chad W. MacPherson[1*], Olivier Mathieu[1], Julien Tremblay[2], Julie Champagne[2], André Nantel[3], Stéphanie-Anne Girard[1] and Thomas A. Tompkins[1]

Supplementary Figures:

Figure S1. Alpha diversity metrics (Shannon index) boxplots computed from all OTUs for each experimental condition. Statistical significance was assessed with a t-test: For each group, comparisons between V3 and V2, V4 and V5 were significant (***: *p*-value < 0.0001 or **: *p*-value < 0.005). Group A - Placebo Supplement; Group B - Probiotic Supplement

Figure S2. Beta diversity (Weighted UniFrac) metrics computed from all OTUs. Each point represents a sample. Permanova was performed using the "adonis" function of the vegan library. Group A - Placebo Supplement; Group B - Probiotic Supplement

Figure S3. Beta diversity (Weighted UniFrac) metrics computed from all OTUs. Each point represents a sample. Permanova was performed using the "adonis" function of the vegan library. In each plot, all points having the same color represent a sample from the same individual for all experimental conditions.

Figure S4. Summary *in silico* analysis of selected enriched ABR genes (Fig.8) for antimicrobial + supplement (visit 3) in both groups from the classes of aminoglycosides and beta-lactams that were linked to various opportunistic pathogens of the family of *Enterobacteriaceae* using the Comprehensive Antibiotic Resistance Database (CARD).

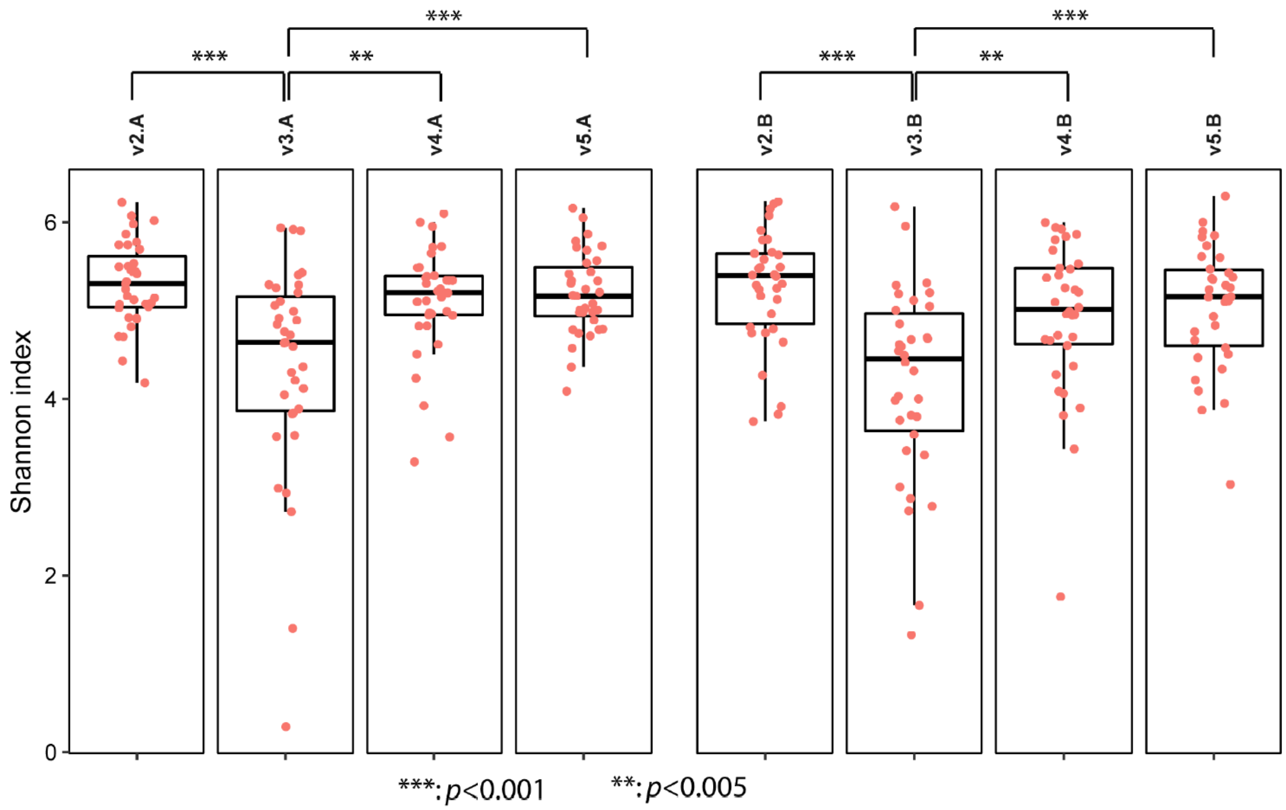

Figure S1. Alpha diversity metrics (Shannon index) boxplots computed from all OTUs for each experimental condition. Statistical significance was assessed with a t-test: For each group, comparisons between V3 and V2, V4 and V5 were significant (***: *p*-value < 0.0001 or **: *p*-value < 0.005). Group A - Placebo Supplement; Group B - Probiotic Supplement

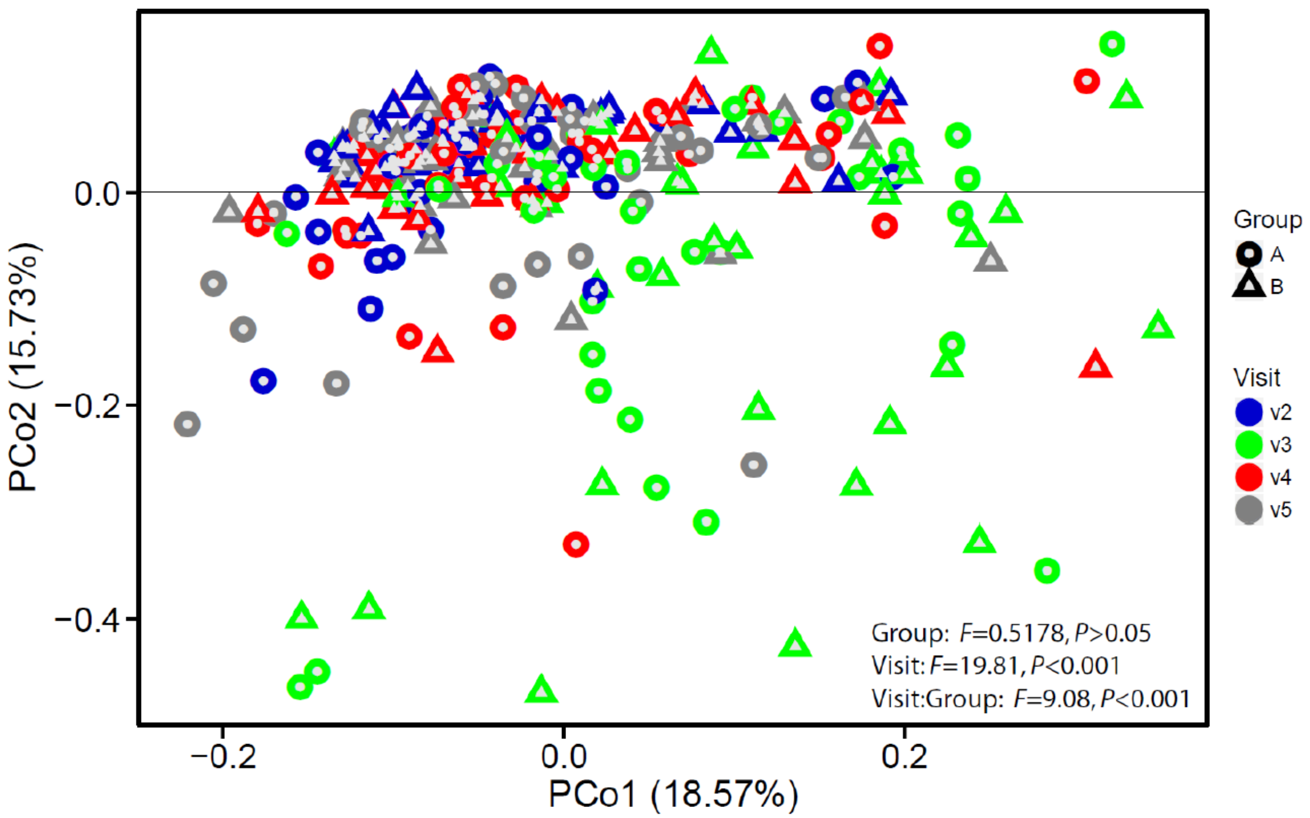

Figure S2. Beta diversity (Weighted UniFrac) metrics computed from all OTUs. Each point represents a sample. Permanova was performed using the "adonis" function of the vegan library. Group A - Placebo Supplement; Group B - Probiotic Supplement

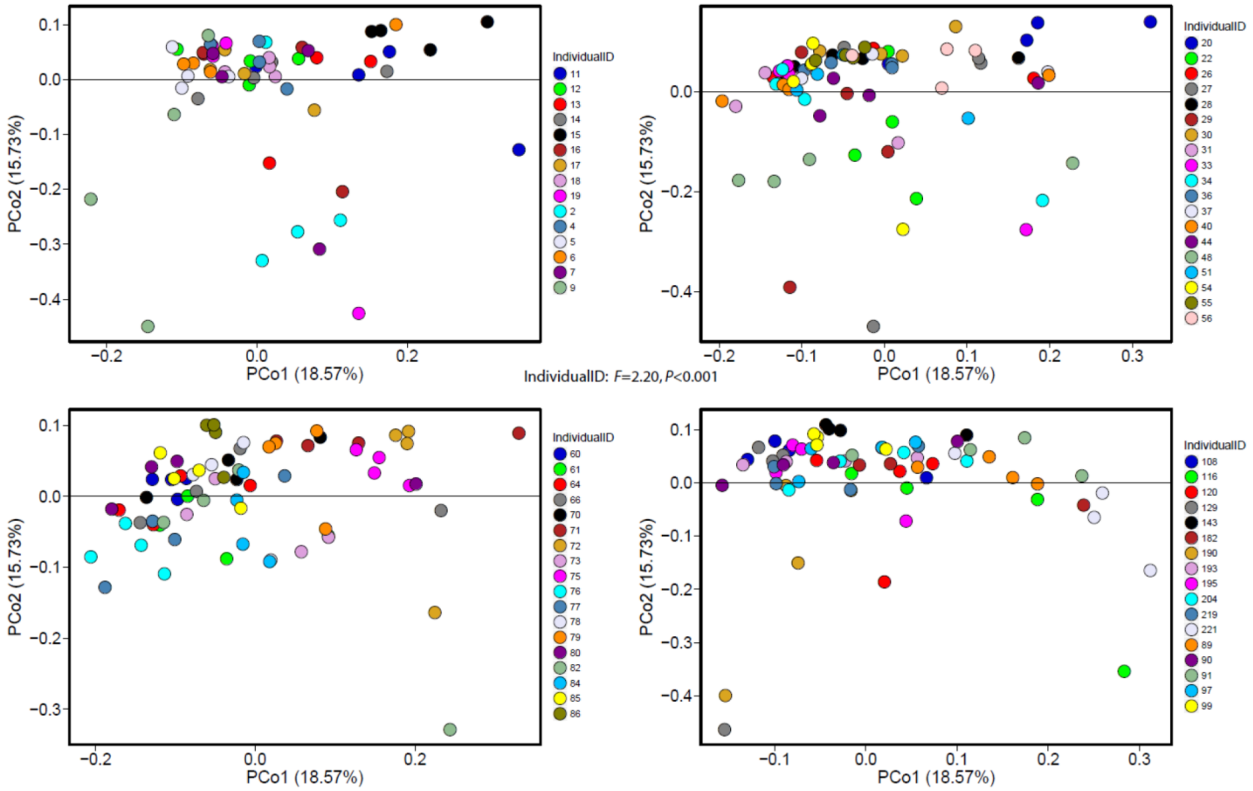

Figure S3. Beta diversity (Weighted UniFrac) metrics computed from all OTUs. Each point represents a sample. Permanova was performed using the "adonis" function of the vegan library. In each plot, all points having the same color represent a sample from the same individual for all experimental conditions.

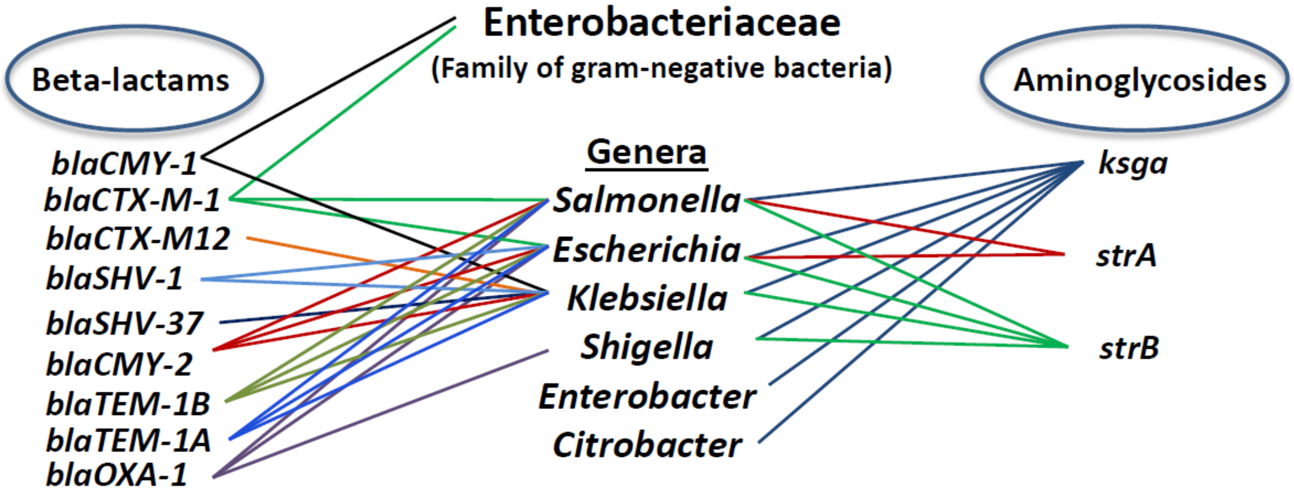

Figure S4. Summary *in silico* analysis of selected enriched ABR genes (Fig.8) for antimicrobial + supplement (visit 3) in both groups from the classes of aminoglycosides and beta-lactams that were linked to various opportunistic pathogens of the family of *Enterobacteriaceae* using the Comprehensive Antibiotic Resistance Database (CARD).
